# Enhanced Skin Delivery of Amphotericin B: Development and Evaluation of Polymeric Nanoparticles Using a Porcine Ear Model

**DOI:** 10.1021/acsomega.5c05091

**Published:** 2025-11-10

**Authors:** Magno Maciel-Magalhães, David Emanuel Ybarra, Daniela Maza Vega, Ayelen Morena Sosa, Beatriz Rodrigues Canabarro, Flavia Fernandes Ferreira da Silva, Helvécio Vinícius Antunes Rocha, Carolina Soledad Martinez, Maria Jimena Prieto, Isabella Fernandes Delgado, Beatriz Ferreira de Carvalho Patricio

**Affiliations:** † Laboratório de Fisiologia, Instituto Nacional de Controle de Qualidade em Saúde (INCQS), 37903 Fundação Oswaldo Cruz (Fiocruz), Av. Brasil, 4365, Rio de Janeiro 21040-900, Brasil; ‡ Programa de Pós-graduação em Pesquisa Translacional em Fármacos e Medicamentos (PPG−PTFM), Fundação Oswaldo Cruz (Fiocruz), Av. Brasil, 4365, Rio de Janeiro 21040-900, Brasil; § Programa de Pós-graduação em Vigilância Sanitária (PPG-VISA), Fundação Oswaldo Cruz (Fiocruz), Av. Brasil, 4365, Rio de Janeiro 21040-900, Brasil; ∥ Laboratorio de Bio-Nanotecnología (LBN), Departamento de Ciencia y Tecnología, 28235 Universidad Nacional de Quilmes (UNQ), Roque Sáenz Peña 352, Bernal, Buenos Aires B1876, Argentina; ⊥ Grupo de Biología Estructural y Biotecnología, Instituto Multidisciplinario de Biología Celular (IMBICE), Consejo Nacional de Investigaciones Científicas y Técnicas, CIC-PBA, UNLP, Calle 526 y Camino General Belgrano, La Plata, Buenos Aires B1906APO, Argentina; # Departamento de Engenharia Metalúrgica e de Materiais, Instituto Alberto Luiz Coimbra de Pós-Graduação e Pesquisa em Engenharia (COPPE), 28125 Universidade Federal do Rio de Janeiro (UFRJ), Av. Horácio Macedo, 2030, Rio de Janeiro 21941-594, Brasil; ∇ Centro Brasileiro de Pesquisas Físicas (CBPF), R. Dr. Xavier Sigaud, 150, Rio de Janeiro 22290-180, Brasil; ○ Laboratório de Micro e Nanotecnologia, Centro de Desenvolvimento Tecnológico em Saúde (CDTS), Fundação Oswaldo Cruz (Fiocruz), Av. Brasil, 4365, Rio de Janeiro 21040-361, Brasil; ◆ Vice-Presidência de Educação, Informação e Comunicação (VPEIC), Fundação Oswaldo Cruz (Fiocruz), Av. Brasil, 4365, Rio de Janeiro 21040-900, Brasil; ¶ Laboratório de Inovação Farmacêutica e Tecnológica, Instituto Biomédico, 89111 Universidade Federal do Estado do Rio de Janeiro (Unirio), R. Frei Caneca, 94, Rio de Janeiro 20211-010, Brasil

## Abstract

Amphotericin B (AmB) is a widely used antifungal drug that is also prescribed to some neglected diseases, such as leishmaniasis. Its usage is limited by its low oral bioavailability and side effects, leading to the exploration of alternative delivery systems. Polymeric nanoparticles (PNPs) have emerged as a promising drug delivery pathway, offering potential benefits such as controlled release and improved drug bioavailability. In this work, AmB-loaded poly­(lactic acid) (PLA) and polycaprolactone (PCL) PNPs were produced by nanoprecipitation and characterized by dynamic light scattering, scanning transmission electron microscopy, and Raman spectroscopy. Subsequently, their stability was tested in static multiple light scattering (SMLS) analysis, and their penetrability was determined in an *ex vivo* porcine skin model. The obtained results indicated that the PNPs were successfully produced. The PLA + AmB PNPs were able to reach the viable epidermis, while the PCL + AmB PNPs permeated the stratum corneum, suggesting that both may be useful for the topical treatment of fungal infections and cutaneous leishmaniasis.

## Graphical abstract

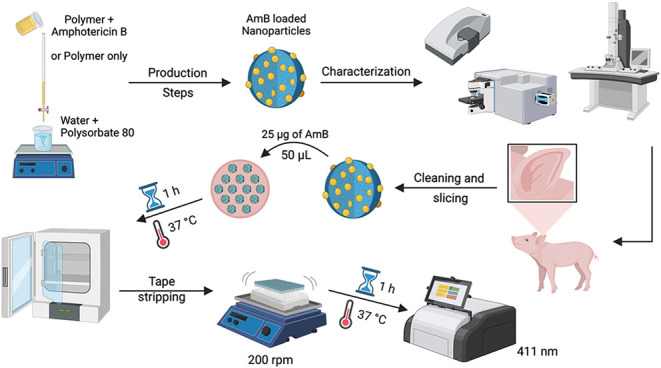

## 1 Introduction

The skin has a complex, multilayered structure. The stratum corneum (SC) is the outermost portion of the skin that serves as the primary barrier to external agents. Measuring approximately 10 to 20 μm, it is composed of approximately 15 to 20 strata of corneocytes, dead cells saturated with keratin, embedded within a hydrophobic environment, rich in lipids such as ceramides. This structure acts as a highly restrictive barrier that prevents the entry of external agents and reduces water loss.^1–3^

However, dermatophytes, a group of filamentous fungi belonging to the genera *Trichophyton*, *Microsporum*, and *Epidermophyton* or yeasts,^4^ possess the unique capacity to metabolize keratin, enabling them to colonize and proliferate within the keratinized tissues of the skin, specifically the hair, nails, and stratum corneum.^5^ These cutaneous fungal infections (CFIs), commonly termed dermatophytoses, are superficial mycoses, but there are other pathogens that can penetrate deeper into the skin layers.

Underlying the SC is the viable epidermis. This avascular tissue is 50 to 100 μm thick, composed of keratinocytes, and divided into four strata: lucid, granular, spinous, and basal or germinative. Due to its nature, systemic absorption of substances rarely occurs. Collectively, the stratum corneum and the viable epidermis form the epidermis, the initial layer of the skin.^2,3,6^

The genus *Candida* belongs to the fungal class *Saccharomycetes* and commonly resides on human skin and mucosal surfaces.^7,8^ These are dimorphic fungi with the filamentous form being responsible for tissue invasion and host-cell damage. In addition to producing lipases and phospholipases, *Candida* secretes aspartyl proteinases, enzymes that hydrolyze glycosidic bonds in proteins, thereby facilitating the fungus’s deeper penetration into the viable epidermis.^9^

The dermis, a 0.5–5 mm thick layer, is a complex connective tissue primarily composed of interstitial components such as collagen, elastic fibers and ground substance, and a variety of cells.^10^ It contains a rich capillary bed, which makes this layer the main site of systemic cutaneous absorption of substances. Consequently, for the successful transdermal administration of drugs, the therapeutic agent must be transported through the epidermis to the superficial dermal capillary bed to be absorbed, which represents a pharmaceutical challenge.^11^

Cutaneous leishmaniasis (CL) establishes itself beyond the protective epidermal barrier, mostly within the dermal layer of the skin. Following transmission by the bite of an infected female sandfly,^12^ *Leishmania* promastigotes are rapidly phagocytosed by host immune cells, predominantly macrophages, where they differentiate into the nonmotile, replicative amastigote stage that multiplies within the macrophage phagolysosomes.^13^ The accumulation of these infected macrophages and other inflammatory cells in the dermis and on the edges of the lesions, thus also being present in the epidermis, form granulomas that characterize the cutaneous wounds,^13–15^ leading to a diverse spectrum of clinical manifestations, ranging from self-healing localized lesions to chronic, disfiguring ulcers, and, in some cases, progression to mucocutaneous leishmaniasis (MCL).^12,14,15^

Amphotericin B (AmB), a macrolide polyene drug used since 1959, is a potent broad-spectrum agent that strongly binds to ergosterol, the primary sterol found in the plasma membrane of both fungal and *Leishmania* parasites.^16^ Transmembrane pores are formed, leading to a critical increase in cellular permeability, loss of cellular integrity, and subsequent death of the pathogens.^16^ Over the years, this pentavalent antimonial has been used to treat local fungal infections and neglected tropical diseases (NTDs).^17^ Despite the potent activity of AmB, its application is challenged by inherent physical–chemical limitations. According to the Biopharmaceutical Classification System, AmB is a Class IV drug. Therefore, it has a low solubility in gastrointestinal fluids and low permeability through the gastrointestinal tract membranes. This profile results in poor oral and topical bioavailability,^18^ consequently, AmB has traditionally been administered via intravenous route.^17^ In efforts to overcome AmB limitations and enhance patient acceptance for the treatment, several pharmaceutical formulations have been developed, but none have successfully avoided intravenous administration.^19^ In recent years, the number of studies applying nanotechnology to develop formulations for alternative routes of AmB administration has significantly increased; however, these efforts have yet to overcome the skin’s intrinsic barrier properties.^20–23^

Polymeric nanoparticles (PNPs) are those consisting of synthetic or natural polymers, usually of small size, and which have attracted the attention of the pharmaceutical industry in recent years due to certain advantages, such as the possibility of designing controlled release models, protection of environmentally sensitive drugs, improvement of their bioavailability, and increasing the therapeutic range generated by reducing toxicity.^24,25^ PNPs offer a promising strategy to enhance and control drug absorption through the skin. This approach improves drug efficacy at target sites, minimizes adverse effects at nontarget areas, reduces systemic absorption, and enhances formulation stability and the therapeutic index of drugs.^26^

Some biodegradable synthetic polymers have been extensively studied in the development of PNPs, such as polyhydroxyesters: poly­(lactic acid) (PLA), polycaprolactone (PCL), poly­(glycolic acid) (PGA), and poly­(lactic-*co*-glycolic acid) (PLGA). In general, these polymers have a favorable safety and biocompatibility profile, low immunogenicity and toxicity, and good biodegradation.^27,28^ Moreover, in recent years, nanotechnologies have been widely explored as an approach to designing delivery systems that are more effective than the vehicles traditionally employed by the pharmaceutical industry for the topical transport of substances through the skin.^29^

Studies on skin penetration are crucial for understanding the effectiveness of drug delivery systems. It is important to elucidate how nanocarriers enhance drug release and to compare the skin penetration profiles of free versus nanoencapsulated drugs.^1,30–32^ These studies can be carried out using the Saarbrücken penetration model.^33–35^ Additionally, porcine skin is frequently employed as a model for human skin due to its comparable morphological and functional characteristics. Even though animal skin generally exhibits higher permeability, research has demonstrated strong *ex vivo* correlations between porcine and human skin for both free and nanoformulated drugs.^1,36–38^ A meta-analysis study has calculated a Pearson correlation coefficient of 0.88 when comparing the *ex vivo* permeability of porcine and human skin.^39^ In this context, the tape stripping method using porcine skin and common adhesive tape has been widely applied to evaluate drug delivery through the skin and to investigate penetration kinetics. This well-established method can be applied both *ex vivo* and *in vivo* and consists of the sequential removal of the stratum corneum.^39^

Thus, with the main goal of developing therapeutic alternatives for parasitic skin infections, especially those caused by fungi and *Leishmania* spp., and in continuation of the previously published work, which elucidated the toxicity and aggregation state of AmB within these PNPs,^40^ the present study aimed to produce and characterize polymeric nanoparticles loaded with Amphotericin B and to investigate their cutaneous penetrability, applying a porcine ear skin model, providing insights regarding their potential applicability.

## 2 Methodology

### 2.1 PNP Production

PNPs were produced by nanoprecipitation, as previously described in Maciel-Magalhães et al. 2025.^40^ Eighty milligrams of the polymer, either PLA (CAS No. 26780–50–7, Mw 18,000–28,000, Sigma-Aldrich) or PCL (CAS No. 24980–41–4, Mw 14,000, Sigma-Aldrich), was solubilized in 15 mL of acetone (CAS No. 67–64–1, Biograde) acidified with 150 μL of 0.1 N HCl (CAS No. 7647–01–0, Biograde). In parallel, the AmB (CAS No. 1397–89–3, Hangzhou Dayangchem) solution was prepared by dissolving 20 mg of the drug in 2 mL of dimethyl sulfoxide (DMSO, CAS No. 67–68–5, Merck), followed by the addition of 5 mL of methanol (CAS No. 67–56–1, Merck). When both solutions were clear, they were mixed, thus forming the OP. The aqueous phase consisted of 60 mL of a 0.3% (w/v) solution of super-refined polysorbate 80 (P80, CAS No. 9005–65–6, Croda) in ultrapure water and was kept at room temperature under constant stirring at 500 rpm on a magnetic plate. The phases were then mixed while maintaining a continuous flow of OP until completely mixed. Then, the mixture was kept under magnetic stirring for 10 min at 500 rpm on the magnetic plate (IKA, RT 15 Power IkaMag, Germany). Subsequently, volatile organic solvents were removed in a rotary evaporator (IKA, RV8, Germany) with the temperature set at 38 °C. The excess of AmB was removed from the PNP suspension by centrifugation (Thermo Fisher Scientific, Megafuge 8), in a 1 h cycle, at 985*g*, at room temperature. Finally, both DMSO and P80 were removed from the final formulation by two ultracentrifugation steps of 15 min each, both at 20,000*g*, 10 °C (Thermo Fisher Scientific, Sorvall MTX150). Subsequently, precipitated nanoparticles were resuspended in ultrapure water and characterized.

### 2.3 PNP Characterization

To determine the concentration of nanoencapsulated AmB, 500 μL of PNP-AmB suspension was transferred to a microtube with a 100 kDa Amicon filter (Merck-Millipore) and centrifuged at 7500*g* for 20 min at room temperature (Thermo Fisher Scientific, Megafuge 8). PNPs were recovered from the filter with 200 μL of a 6:4 acetonitrile (ACN):DMSO mixture and diluted in a volumetric flask. Readings were performed in a spectrophotometer at a wavelength of 411 nm, followed by the necessary calculations. PNP hydrodynamic diameter, polydispersity index (PdI), and ζ-potential were obtained by dynamic light scattering (DLS) with a He–Ne laser (λ = 633 nm) and a 90° fixed-angle detector (Malvern Zetasizer Nano ZS90, U.K.). For these analyses, ultrapure water was used as a dispersant, and measurements were performed in triplicate with 15 runs of 10 s.

To determine the PNP shape and dry size, samples were diluted in ultrapure water to achieve a concentration of 50 ng/mL AmB, dispersed using an ultrasonic bath (UltraCleaner 1400, Unique, Brazil) and subsequently dropped onto 200 mesh Lacey Formvar/Carbon copper grids (Ted Pella). The analysis was performed by scanning transmission electron microscopy with a high-angle annular dark field detector (STEM-HAADF) on a Talos F200X (Thermo Fisher) operating at 200 kV.

To verify interactions between the drug and the polymers, Raman spectroscopy was applied as follows. The samples were deposited on the specific sample holder for powders, lightly pressed, and read on an FT-Raman spectrophotometer (MultiRAM, Bruker, Germany) with a laser power of 200 mW and wavelength of 1064 nm, resolution of 4 cm^–1^, 200 accumulated scans, and in the range between 3 and 3600 cm^–1^.

### 2.4 PNP Stability Studies

Particle sedimentation was observed using the static multiple light scattering (SMLS) technique with Turbiscan Lab (Microtrac). The analysis was performed at 27 °C and initiated right after the preparation of the PNP suspensions. The samples were tested as suspensions in ultrapure water, with a pH value of approximately 5.5, and with the pH adjusted to 7.4. Scan readings were taken with the following analysis sequence: scans every 30 s in the first hour (121); scans every 5 min in the following 4 h 30 mn (49); scans every 30 min for the following 20 h (39); and scans every hour in the remaining 6 days (145), totaling 168 h (7 days).

### 2.5 Skin Penetrability Evaluation

The porcine ears used in this test were purchased from a commercial cold storage facility located in Quilmes, Argentina. First, the skin was excised from pig ears, and the subcutaneous tissue was removed prior to storage at −20 °C. On the day of usage, skin disks measuring 2.5 cm in diameter and 0.28 cm in thickness, corresponding to an area of 4.9 cm^2^ and a volume of 1.37 cm^3^, were prepared. The disks were placed on Teflon supports (Saarbrücken Penetration Model devices). A total of 50 μL of each sample was applied in concentric drops. Samples of PCL or PLA PNP containing AmB, as well as free AmB solubilized in 1% DMSO, were seeded. Each disk received a total of 25 μg of AmB. Distilled water was seeded in a fourth skin disk as the negative control. The treated skin disks were incubated in an oven for 1 h at 37 °C and then gently cleaned with cotton to remove unpenetrated material and secured to a support using pins. The tape stripping protocol was carried out as described by Izquierdo et al.^1^ Briefly, adhesive tape was applied to the skin with a force of 19.6 N (2 kg) for 10 s and removed using tweezers. The adhesive strips, in sequential sets of five, containing the *stratum corneum*, as well as the remaining skin (composed of viable epidermis and dermis) and the cotton used for cleaning, were placed in 15 mL tubes. AmB was extracted using 2 mL per tube of a 6:4 ACN:DMSO mixture for 1 h at 37 °C and under constant stirring at 200 rpm. The concentration in each tube was determined using a spectrophotometer (Shimadzu, UV-1603, Japan) at 411 nm. The values obtained from negative control skin strips were used as the background and subtracted from the correlated strips from other samples. Tape stripping was performed in quadruplicate.

The skin penetration profile was further assessed via epifluorescence microscopy. After the incubation period, the disks were frozen at −20 °C, embedded in an Optimal Cutting Temperature compound (OCT, Sigma-Aldrich), and sectioned into 15 μm transverse slices using a Leica CM 1850 cryomicrotome (Leica, Germany). Images were acquired in a Cytation 5 (BioTek/Agilent), applying the fluorescence microscopy functionality with a GFP filter (excitation, 469 em., 525 nm) at 25× magnification.

### 2.6 Statistical Analysis

Statistical analyses were performed with GraphPad Prism 8.3.1 for macOS. All data were evaluated for normality using the Kolmogorov–Smirnov test, and values >0.05 were considered normal. Subsequently, two-way analysis of variance (ANOVA) was applied. The result was considered statistically different from the control group when *p* < 0.05, followed by Tukey’s multiple comparison test.

## 3 Results and Discussion

### 3.1 PNP Characterization

Using the obtained STEM-HAADF images (Figure 1), PNP diameters were determined: poly­(lactic acid) nonloaded nanoparticles (PLA NL) 26.23 ± 3.64 nm; poly­(lactic acid) nanoparticles loaded with AmB (PLA + AmB) 38.21 ± 12.05 nm; polycaprolactone nonloaded nanoparticles (PCL NL) 73.79 ± 28.74 nm; and polycaprolactone nanoparticles loaded with AmB (PCL + AmB) 93.07 ± 13.18 nm.

**Figure 1:**
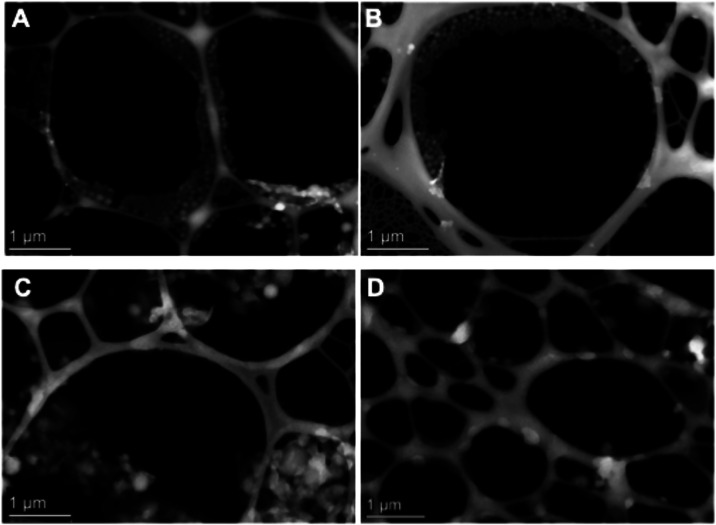
Scanning transmission electron microscopy with a high angular annular dark field detector (STEM-HAADF) image of the polymeric nanoparticles: (A) Nonloaded nanoparticles of poly­(lactic acid); (B) nanoparticles of poly­(lactic acid) loaded with amphotericin B, (C) nonloaded nanoparticles of polycaprolactone, and (D) nanoparticles of polycaprolactone loaded with amphotericin B.

Their hydrodynamic diameters, PdI, and ζ-potential values, obtained by the DLS technique, are shown in Table 1. As can be seen in both the STEM images and DLS measure, the PLA nanoparticles demonstrated a tendency to be smaller in size than the PCL ones, and there was an increase in the diameter of both after AmB encapsulation, suggesting that the drug was indeed loaded onto the PNP.

**Table 1:** Dynamic Light Scattering Results of Size (nm), Polydispersity Index, ζ-Potential (mV), and Amphotericin B Concentration Analysis of Polymeric Nanoparticles Nonloaded and Loaded with Amphotericin B^a^

	NL PNP			PNP loaded with AmB			
	size (nm)	PdI	ζ-potential (mV)	size (nm)	PdI	ζ-potential (mV)	[AmB] (μg/mL)
PLA	134.4	0.179	–30.8	205.1	0.160	–25.5	181.54
PCL	183.7	0.070	–29.1	227.7	0.093	–19.1	150.87

a PdI: polydispersity index; PLA: poly­(lactic acid); PCL: polycaprolactone; PNP: polymeric nanoparticles; NL: nonloaded; AmB: amphotericin B. Analysis carried out at 25 °C, with a He–Ne laser (λ = 633 nm) and a fixed-angle detector at 90°. Nanoparticles were suspended in ultrapure water.

The differences in particle size results between STEM-HAADF and DLS techniques may be due to the fact that DLS measures the hydrodynamic size of particles, while STEM measures dry PNP. This explains why DLS yielded larger particle sizes (Table 1) compared to the individual analysis performed using the micrographs. If we analyze the PdI, which gives us an idea of the uniformity of the samples, a PdI close to zero indicates a homogeneous sample, while a PdI close to 1 indicates a heterogeneous sample with different particle size populations.^41,42^ All samples obtained a PdI close to 0 (values between 0.07 and 0.179), indicating that they have homogeneous particle size distributions.

Regarding the ζ-potential, it is noticeable that both PNPs had a decrease in the obtained values when AmB was added to the system. Therefore, we may suggest that AmB could be located, at least partially, on the surface of the nanoparticles, modifying their superficial electrostatic charge.

### 3.2 PNP Raman Spectroscopy

Spectroscopies are appropriate tools to characterize the interaction between the components of a nanoformulation.^43^ In particular, Raman spectroscopy can detect the vibrational modes of molecules and their Raman shifts, even in aqueous suspensions. Figure 2A,B displays the Raman spectra of free AmB and its characteristic bands at 1001.08, 1153.13, and 1557.98 cm^–1^. The band at 1001.08 cm^–1^ is assigned to C–C and C–H in-plane bending vibrations of aromatic ring structures.^44^ The intermediate-intensity band at 1157.98 cm^–1^ is associated with aromatic ring deformations and C–H bending modes.^44^ The most intense band, located at 1557.98 cm^–1^, corresponds to the C=C stretching vibrations within the conjugated polyene region of AmB, indicative of its resonance structure.^44,45^

**Figure 2:**
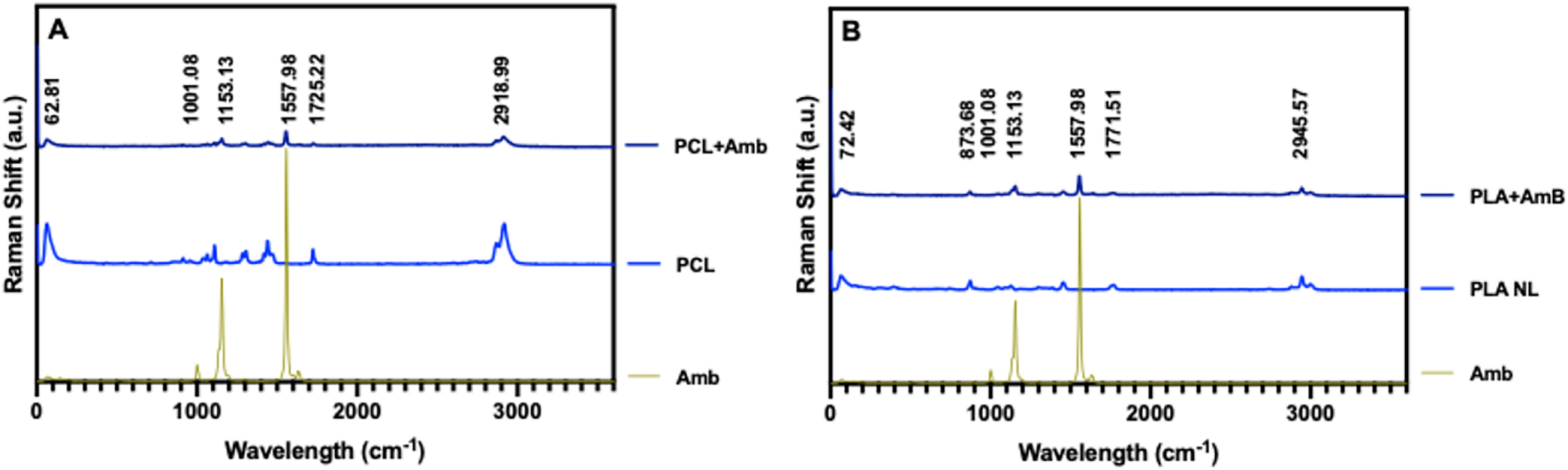
Raman spectra of (A) nonloaded nanoparticles of polycaprolactone (PCL NL) and loaded with AmB (PCL + AmB) and (B) nonloaded poly­(lactic acid) nanoparticles (PLA NL) and loaded with AmB (PLA + AmB). In both graphs, free amphotericin B is also shown.

Figure 2A also presents the Raman spectra of PCL formulations with and without AmB. In the spectrum of PCL NL PNP, characteristic bands are observed at 62.81, 1725.22, and 2918.99 cm^–1^. The low-frequency band at 62.81 cm^–1^ is attributed to collective vibrational modes, such as torsional or vibrational motions of the polymer chains, and may also reflect interlamellar vibrations. The intense band at 2918.99 cm^–1^ corresponds to the asymmetric stretching vibration of the methylene groups (−CH_2_−), a typical feature of aliphatic polyesters.^46^ The band at 1725.22 cm^–1^, corresponding to the C=O stretching vibration of the ester groups, is a key spectral marker in Raman spectroscopy for polyesters due to its high sensitivity to local molecular environments.^47,48^ In the PCL + AmB formulation, AmB characteristic bands appear at 1154.37 and 1557.44 cm^–1^, while PCL bands exhibit notable attenuation, suggesting molecular interactions between AmB and PCL chains, such as hydrogen bonding or hydrophobic effects, which can disrupt the regular packing of the polymer chains and induce conformational disorder. These findings are consistent with a partial structural reorganization of the PCL matrix upon incorporation of the drug.

On the other hand, Figure 2B presents the Raman spectrum of PLA nanoparticles, in which characteristic bands are observed at 72.45, 873.68, 1771.51, and 2945.57 cm^–1^. The low-frequency band at 72.45 cm^–1^ is attributed to collective vibrational modes, including torsional or vibrational motions of the polymer backbone. The band at 873.68 cm^–1^ corresponds to out-of-plane deformation vibrations of the −CH– group associated with vibrational modes preserved from the structural features of the lactide monomer. The high-frequency band at 2945.57 cm^–1^ is assigned to the asymmetric stretching of C–H bonds in methyl (−CH_3_) and methylene (−CH_2_−) groups, a typical feature of aliphatic polymers such as PLA.^49–51^ In amorphous PLA, the C=O stretching is observed at higher wavenumbers (typically 1766–1771 cm^–1^) than in semicrystalline PCL due to the lack of ordered chain packing and the electron-withdrawing effect of α-methyl groups, which strengthens the C=O bond. The absence of bands at 922 and 540 cm^–1^, associated with helical conformation of crystals, also suggests the amorphous state of PLA.^52,53^ Upon the incorporation of AmB, the characteristic PLA bands are attenuated, presumably due to physical dispersion of the drug within the polymer matrix and possible molecular interactions affecting polarizability, while distinct AmB signals became evident.

In summary, these results confirm the successful incorporation of AmB into PCL and PLA-based nanoparticles, as evidenced by the appearance of Raman bands characteristic of AmB and the corresponding attenuation of the signals from the corresponding native polymers, corroborating our suggestion regarding the ζ-potential.

### 3.3 PNP Stability Studies

The stability analysis of amphotericin B-loaded polymeric nanoparticles (PNP + AmB) using the SMLS Turbiscan technique revealed sedimentation in all tested formulations. However, this phenomenon was more pronounced in PLA nanoparticles compared to those made with PCL with or without AmB (Figures 3 and 4). After 7 days, the cumulative sedimentation of PLA + AmB PNP was approximately 1.9 times higher than that of PCL + AmB formulations, indicating the lower physical stability of the PLA matrix (Figure 3). In contrast, a reduction in backscattering (BS) was observed in the central region for PCL samples, suggesting an increase in particle size, an observation further corroborated by DLS measurements (Table 2). Compared to the blank nanoparticles, those loaded with AmB exhibited increased instability (Figure 4), which correlates with the increase in the hydrodynamic size (Table 1). This finding is consistent with the expected destabilizing effect of drug encapsulation on nanoparticle systems.^54^ Nevertheless, the observed instability remained below a 5-fold increase, which is not substantial enough to justify discarding the samples

**Figure 3:**
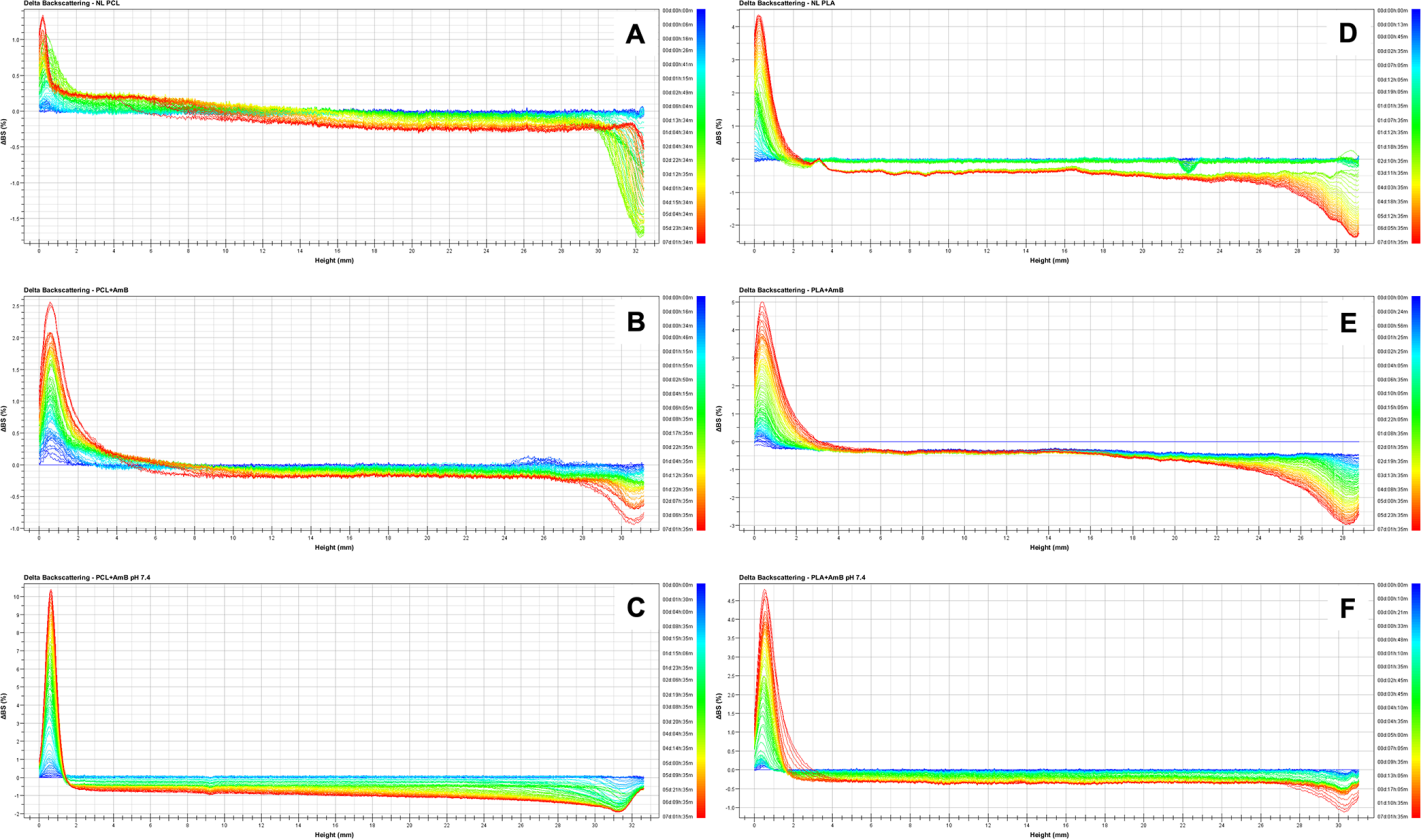
Backscattering (ΔBS) profiles obtained through the Turbiscan analysis for polymeric nanoparticle formulations containing amphotericin B (AmB): (A) NL PCL, (B) PCL + AmB, and (C) PCL + AmB at pH 7.4, (D) NL PLA, (E) PLA + AmB, and (F) PLA + AmB at pH 7.4. Progressive sedimentation over time is observed in all samples, with the effect more pronounced at higher pH values.

**Figure 4:**
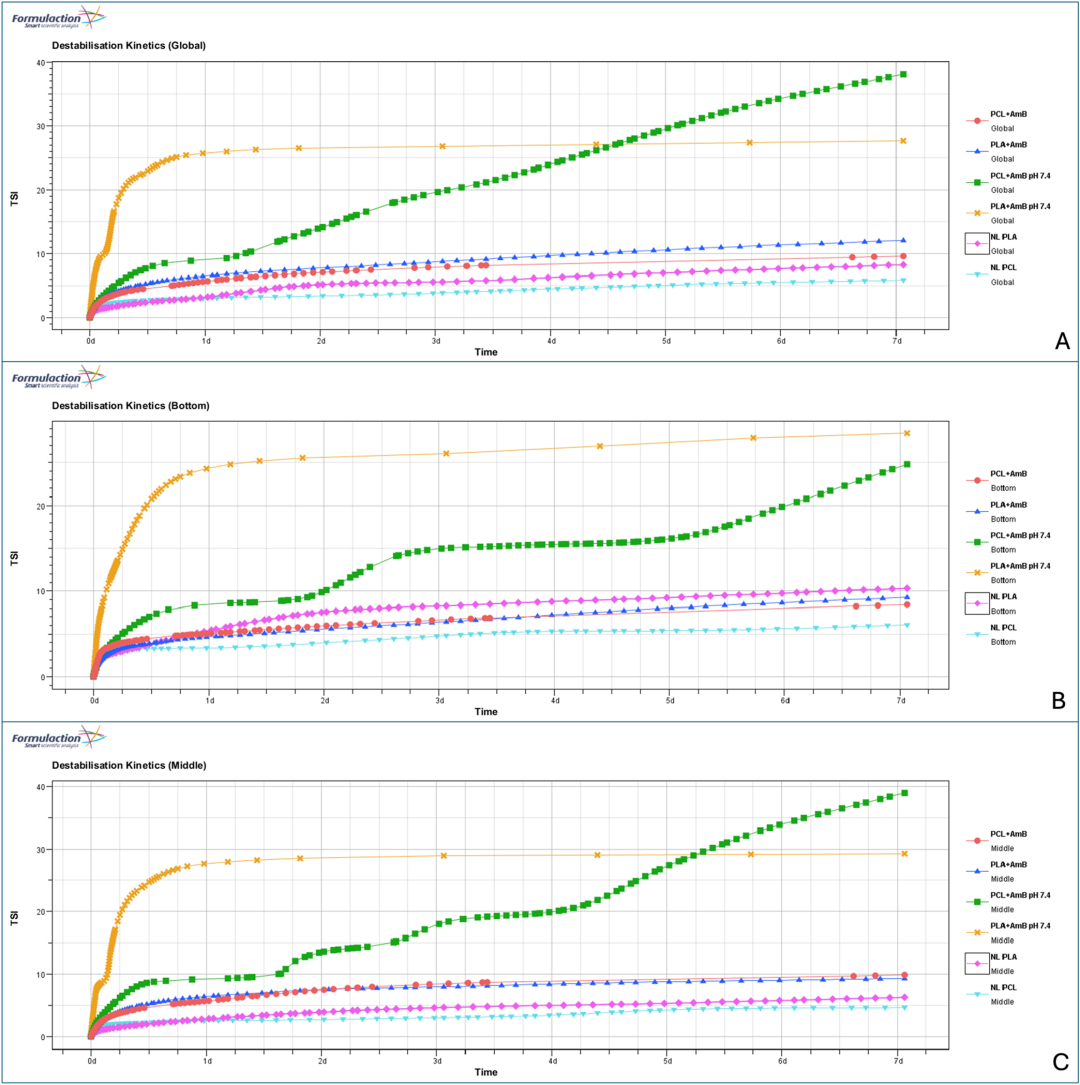
Turbiscan stability index (TSI) values obtained for different zones of the sample over time: (A) global TSI; (B) TSI in the bottom region; and (C) TSI in the middle region. Distinct destabilization patterns can be observed among the formulations, with a marked progressive instability for PCL nanoparticles at pH 7.4.

**Table 2:** Size and PdI of the Nanoparticles Determined by DLS at Time 0 and 7 Days

	PCL + AmB		PLA + AmB		PCL + AmB pH 7.4		PLA + AmB pH 7.4	
time (days)	size (nm)	PdI	size (nm)	PdI	size (nm)	PdI	size (nm)	PdI
0	178.4	0.159	182.3	0.174	305.2	0.343	118.3	0.229
7	191.8	0.083	182.8	0.162	627.7	0.522	113.7	0.186

The influence of the pH on stability was evident. At pH 7.4, the PCL nanoparticles exhibited an increased size and PdI even at time zero (Table 2). SMLS data showed a marked reduction in BS in the central region of the sample after just 24 h, suggesting a sedimentation rate approximately 4 times higher than that observed under acidic conditions (Figure 3). This behavior was further supported by the TSI data, which indicated a progressive and almost linear destabilization from 1 day and 4 h onward (Figure 4).

For PLA nanoparticles at pH 7.4, a uniform decrease in BS in the central zone was observed, suggesting particle aggregation (Figure 3). Aggregation likely accelerated sedimentation, as demonstrated by the early TSI plateau, which was reached after approximately 1 day and 4 h (Figure 4). This finding was corroborated by DLS measurements taken at different heights along the tube, which revealed increased particle sizes at the bottom and heterogeneous populations, as indicated by the higher PdI values (Table 3). However, these particles were readily redispersed, as noted in the average particle size at day 7, which remained comparable to the original measurement, suggesting good recuperation of the system (Table 2).

**Table 3:** Size and PdI of Nanoparticles after 7 Days of Turbiscan Analysis, Sampled from the Middle and Bottom of the Tube

	PCL + AmB		PLA + AmB		PCL + AmB pH 7.4		PLA + AmB pH 7.4	
tube high	size (nm)	PdI	size (nm)	PdI	size (nm)	PdI	size (nm)	PdI
middle	191.5	0.110	174.1	0.128	357.63	0.321	106.40	0.061
botton	201.1	0.118	181.8	0.152	628.83	0.471	207.73	0.648

Overall, increasing the pH resulted in pronounced instability in both formulations, although the mechanisms and dynamics of destabilization differed between them. Zone-specific TSI analysis showed persistent sedimentation regardless of polymer type or pH, but with faster kinetics under basic conditions (Figure 4). In the central region, destabilization was already evident under acidic conditions by day 1, followed by a plateau on the second day, indicating the initial formation of particle aggregates. This phenomenon was exacerbated at higher pH, particularly for the PLA-based system, which reached an instability plateau more rapidly and at higher TSI values, likely due to agglomerate formation.

In contrast, PCL nanoparticles at pH 7.4 showed instability characterized by multiple TSI steps without reaching a plateau, suggesting continuous aggregation and sedimentation (Figure 4). After 4 days, the TSI of PCL nanoparticles exceeded that of PLA formulations at the same pH, possibly due to secondary aggregation and formation of denser sediment structures. This observation was corroborated by DLS, which showed a 2-fold increase in particle size at the bottom compared to the initial size (Table 3). Furthermore, the global DLS measurements showed an increase in particle size from day 0 to day 7, indicating poor redispersibility and potential coalescence, which is likely accelerated by the alkaline pH (Table 2).

Taken together, these results suggest that PCL nanoparticles are generally less stable than PLA nanoparticles containing AmB and that pH control is crucial regardless of the polymer used. This pH range was selected based on literature reports indicating that AmB is more stable between pH 5 and 7.^55,56^ Additionally, pH has a significant impact on the release and stability of AmB in polymeric nanoparticle systems, with greater drug release observed under neutral pH conditions.^57^ Previous studies have demonstrated that nanoparticle stability is significantly influenced by the dispersion medium’s pH, which affects surface charge, AmB solubility, and aggregation potential.^40,57–59^ Moreover, the use of Turbiscan to monitor nanoparticle stability has been validated in recent literature, demonstrating its capability to detect early sedimentation and flocculation phenomena in colloidal dispersions.^60^ Additionally, differences in crystallinity and drug–polymer interactions between PLA and PCL may explain the divergent stability profiles observed.^40,57^

### 3.4 Skin Penetration of AmB-Loaded PNP versus the Free Drug

Using the tape stripping technique, it was possible to observe that the PLA + AmB PNP penetrated the porcine ear skin more efficiently, reaching the “viable epidermis” with a high content (26.12 ± 8.99%). The PCL + AmB PNP demonstrated lower penetration efficiency, overcoming the initial layer of the *stratum corneum*, but only a small amount (2.55 ± 5.10%) reached the viable epidermis. Most of the free AmB (35.56 ± 5.36%) was removed during the cleaning process with cotton prior to tape stripping (Figure 5), showing that the free drug cannot penetrate the *stratum corneum.*

**Figure 5:**
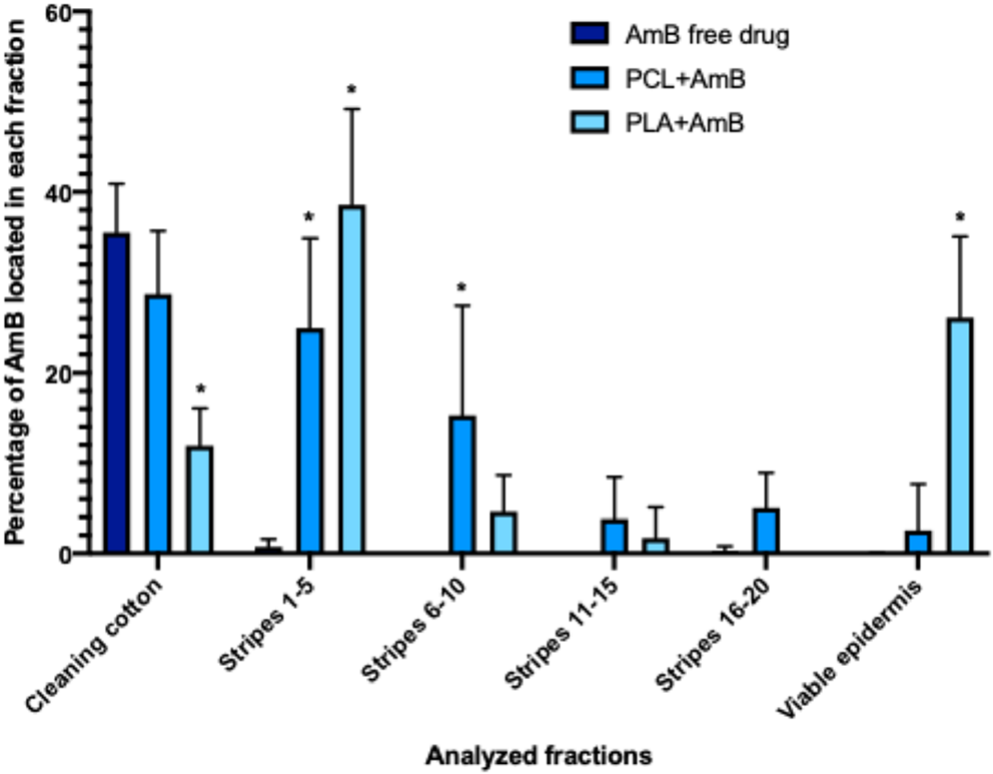
Percentage of AmB in each fraction analyzed by a tape stripping test. PCL, polycaprolactone; PLA, poly­(lactic acid); and AmB, amphotericin B. Assays were performed in quadruplicate. The data is presented as mean ± standard deviation. *Significant difference in relation to nonencapsulated AmB (*p* > 0.05).

Fluorescence microscopy corroborated these findings. Given that AmB is excitable at 408 nm and emits fluorescence at approximately 560 nm,^61^ the GFP filter was applied, which best approximated these theoretical values, to observe the presence of AmB in the skin layers. These micrographs are shown in Figure 6.

**Figure 6:**
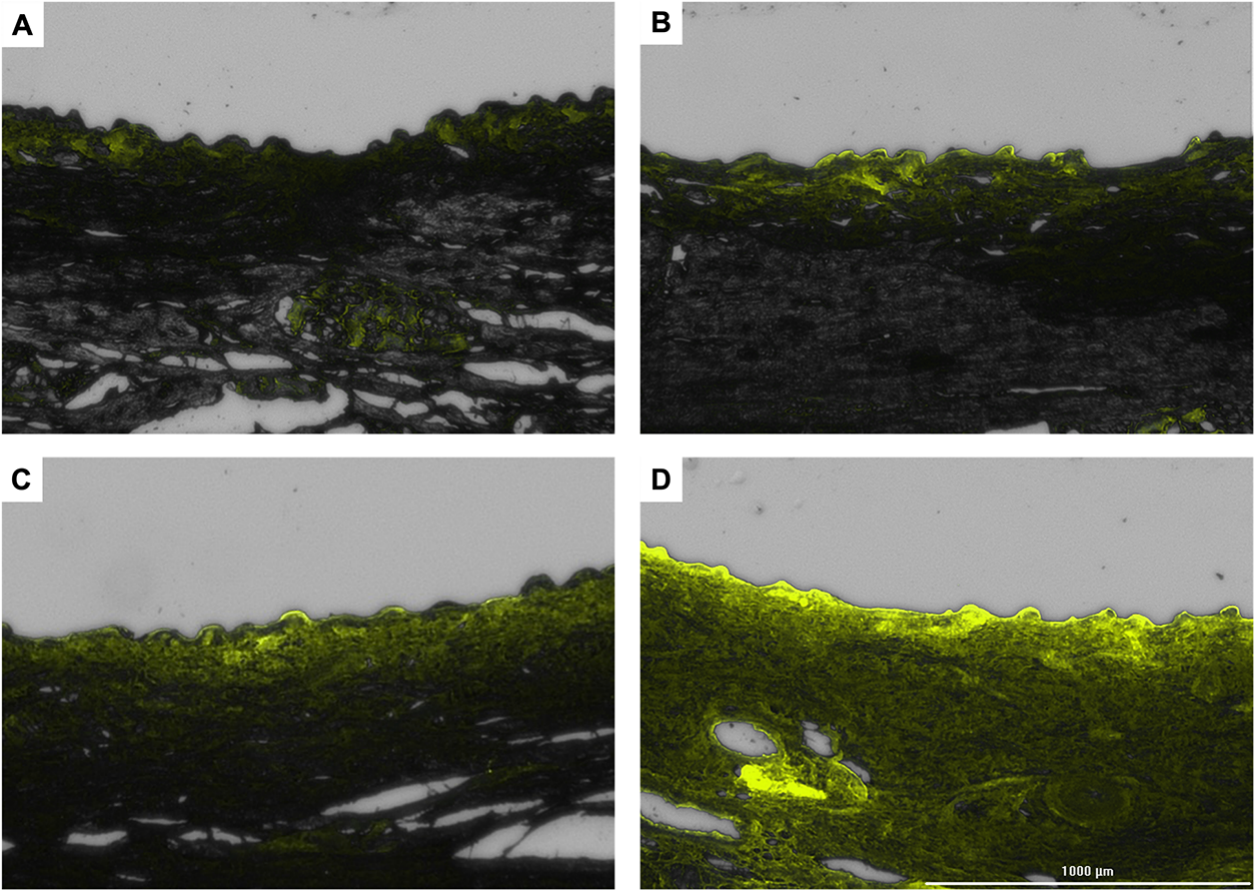
Fluorescence micrographs (*E*_x_: 469 nm; *E*_m_: 525 nm; 25× magnification) of porcine ear skins exposed to PCL or PLA PNP containing AmB or the free drug: (A) Negative control, (B) nonencapsulated AmB, (C) PCL + AmB, and (D) PLA + AmB. The three samples were tested at the same concentration of 500 μg/mL. PNP, polymeric nanoparticles; PCL, polycaprolactone; PLA, poly­(lactic acid); AmB, amphotericin B; and FD, free drug.

Skin treated with PLA + AmB PNP exhibited higher fluorescence intensity than the others, as well as deeper penetration when compared to PCL + AmB PNP and free AmB. These distinct penetration profiles are of great scientific interest and can be rationalized by considering the physicochemical properties of the constituent polymers. Polycaprolactone (PCL) is known to be a hydrophobic polymer due to its 6-carbon-long hydrocarbon chain per repeating unit^62,63^ and probably forms strong hydrophobic interactions with the lipids in the *stratum corneum*, leading to PCL+AmB PNP entrapment into this layer and restricted skin penetration. It should be emphasized that PCL PNP showed superior long-term stability, and the hydrophobicity of this polymer could be beneficial for AmB’s sustained release *in loco*,^64^ which can be an interesting attribute for the topical treatment of dermatophytoses.

Poly­(lactic acid) (PLA), although still a hydrophobic polymer, possesses a relatively greater presence of oxygen atoms and a shorter hydrocarbon chain per repeating unit when compared to PCL,^65^ resulting in PLA being more polar and slightly more hydrophilic and having a direct influence on its surface properties.^66,67^ This balance between PLA hydrophobicity and hydrophilicity may be responsible for the initial interaction with the SC and subsequent permeation of PLA + AmB PNP into the viable epidermis. The presence of PLA nanoparticles within this layer highlights their promising applicability for the treatment of epidermal fungal infections, including *Candida* species that reside in the viable epidermis.

In a study published in 2020, Fernández-Garcia and collaborators^68^ used an *in vivo* tape stripping technique to test the penetrability of ultradeformable lipid vesicles (transferosomes) containing AmB, measuring approximately 150 nm. AmB was diluted only in DMSO, a known permeability-enhancing agent, and researchers detected the presence of AmB in the viable dermis. It is worth mentioning that in our research, free AmB was previously solubilized in DMSO and then diluted to the desired concentration (500 μg/mL) with distilled water, reaching a final DMSO concentration of 1% v/v. This dilution in water was done to mitigate the permeation enhancer effect of DMSO.^69^

Regarding the transferosomes in Fernández-Garcia and collaborators,^68^ they were also observed to penetrate the skin, similar to the results achieved with PLA + AmB PNP, demonstrating that PLA formulations can perform comparable penetrations without relying on the high concentrations of chemical permeation enhancers.

Other studies have already developed AmB-loaded nanoformulations, such as microemulsions, that have proved effective in the treatment of vaginal candidiasis. In one investigation, researchers evaluated two topical AmB formulations in an *in vivo* model of vaginal candidiasis using BALB/c mice. A conventional cream was administered once a day for six consecutive days, while a microemulsion, which underwent *in situ* transformation into a transparent gel, was applied three times at 48 h intervals. The results revealed that microemulsion was more efficient in reducing the fungal colony than the traditional cream, while also decreasing frequency of applications, which improved treatment adherence.^70^

However, not all microemulsion research involving AmB encapsulation achieved permeation into the viable epidermis. For example, the study by dos Santos Matos and collaborators^61^ reported nanoemulsions with controlled release, reduced cytotoxicity, and a more prominent Leishmanicidal effect when compared to free AmB. However, the systems were not able to penetrate the skin, which the authors attributed to the high hydrophobicity of the formulation. Theoretically, these systems would only be effective for treating dermatophytoses in the *stratum corneum*, much like the present PCL + AmB PNPs.

Another study prepared PNP from poly­(lactic-*co*-glycolic acid) (PLGA), a different lactide-based polyester,^71^ loaded with AmB, with the intention of reducing drug toxicity and facilitating localized delivery over a prolonged time. In an *in vivo* experiment performed with BALB/c mice, the authors demonstrated that a single intralesional administration to infected BALB/c mice revealed that these PNPs were more effective than AmB deoxycholate in terms of reducing lesion area in cases of cutaneous leishmaniasis.^72^ This finding leads us to believe that the use of polymeric nanoparticles, such as those studied in both the article by Ammar et al. and in the present study, is useful to improve the applicability of AmB in topical treatments.

However, not all PNPs are suitable for the topical treatment of *Leishmania*. The polymers chosen for the present study performed better in penetrating the skin than chitosan, for example. Riezk et al.^73^ employed this natural polymer in the production of two distinct nanoparticles containing AmB, one positively charged with tripolyphosphate sodium (size = 69 ± 8 nm, ζ-potential = 25.5 ± 1 mV) and another negatively charged with dextran sulfate (size = 174 ± 8 nm, ζ-potential = −11 ± 1). Although both demonstrated activity against *Leishmania major* amastigotes, neither showed good cutaneous permeation, leading the researchers to conclude that they were not good candidates for topical treatments. Considering the hydrophilic and mucoadhesive nature of chitosan,^74^ it is plausible to suggest that hydrophobic PLA and PCL interact better with cutaneous tissues and, therefore, are more appropriate for the dermal nanodelivery of AmB.

It is worth mentioning that, although experimental results obtained *in vitro* or *ex vivo*, such as the excised skin tests, remain of considerable importance, evidence suggests that, under *in vivo* conditions, penetration rates may be up to 10 times higher than those measured by these methods.^75,76^ This suggests that both PNPs presented in this work could be efficacious against pathogens located in the deeper skin layers.

Although we cannot guarantee, with the skin experiments carried out to date, that the PNPs did penetrate the dermis, the results found were promising in this regard, especially when observed together with those published in Maciel-Magalhaes et al.^40^ In this study, our group demonstrated that the nanoparticles containing AmB were able to penetrate the body of zebrafish larvae at a developmental stage in which they do not yet open their mouths, leading to the assumption that the PNPs penetrated through their skin. Finally, it is worth mentioning that we have also demonstrated, in the same work, that PLA + AmB PNP penetrated the animals’ bodies with greater efficiency than PCL + AmB PNP,^40^ same as observed in the present study, in the pig ear skin model. Combining the group’s two findings, as next steps, we believe that PLA + AmB and PCL + AmB PNP should advance to mammals’ *in vivo* experiments, with the aim of confirming their transcutaneous penetrability and performing pharmacokinetic tests based on cutaneous absorption.

## 4 Conclusions

The production of PCL and PLA PNP loaded with AmB occurred as desired, generating spherical nanoparticles slightly larger than their unloaded counterparts, suggesting that the drug encapsulation occurred as expected. Using the *ex vivo* porcine ear skin model, it was possible to determine that both PNPs demonstrated superior permeation compared with the free AmB. PLA + AmB PNP penetrated down to the viable epidermis layer, while PCL + AmB PNP was less efficient, being retained within the *stratum corneum*. These findings, together with supporting evidence from the literature, indicate that the nanosystems developed here, particularly PLA-based formulations, represent promising candidates for the topical treatment of infectious diseases across different skin layers, including dermatophytosis, candidiasis, and, potentially, cutaneous leishmaniasis. Finally, we believe that follow-up studies, including PLA–PCL blend systems, as well as formulating these systems into creams, ointments, or gels in order to test their *in vivo* pharmacokinetics, will be a good idea for future work.

## ⋈

Instituto Nacional de Controle de Qualidade em Saúde, Departamento de Farmacologia e Toxicologia, Av. Brasil, 4365, Manguinhos, Rio de Janeiro 21040-900, Brasil

## Abbreviations

ACN: acetonitrile
AmB: amphotericin B
DLS: dynamic light scattering
DMSO: dimethyl sulfoxide
NL-PNP: nonloaded polymeric nanoparticles
NM: nanomaterials
OP: organic phase
P80: polysorbate 80
PCL: polycaprolactone
PCL + AmB: polycaprolactone nanoparticles loaded with AmB
PdI: polydispersion index
PGA: poly­(glycolic acid)
PLA: poly­(lactic acid)
PLA + AmB: poly­(lactic acid) nanoparticles loaded with AmB
PLGA: poly­(lactic-*co*-glycolic acid)
PNP: polymeric nanoparticles
PNP + AmB: polymeric nanoparticles containing amphotericin B
TEM: transmission electron microscopy
